# Implication of Gut Microbiota in Nonalcoholic Fatty Liver Disease

**DOI:** 10.1371/journal.ppat.1004559

**Published:** 2015-01-27

**Authors:** Jerome Boursier, Anna Mae Diehl

**Affiliations:** 1 Service d’Hépato-Gastroentérologie, Centre Hospitalier Universitaire d’Angers, Angers, France; 2 HIFIH, UPRES 3859, SFR 4208, Université LUNAM, Angers, France; 3 Division of Gastroenterology, Department of Medicine, Duke University Medical Center, Durham, North Carolina, United States of America; Geisel School of Medicine at Dartmouth, UNITED STATES

It is now well established that gut flora and chronic liver diseases are closely interrelated. This association is most evident at late stages of the disease: cirrhosis and impaired liver function are associated with intestinal bacterial overgrowth, small bowel dysmotility, increased gut permeability, and decreased immunological defenses, all of which promote bacterial translocation from the gut to the systemic circulation, leading to infections that in turn aggravate liver dysfunction in a vicious circle [1]. For a long time, the implication of gut flora in the pathophysiology of less advanced chronic liver diseases has been underestimated because technical limitations allow only for the culture of a small fraction of gut bacteria. Recent technological progress and next-generation DNA sequencing have allowed for more sophisticated analysis and sampling of the gut microbiota by culture-independent methods [2]. Thanks to these recent technological advances, knowledge about the role of gut microbiota disruption (dysbiosis) in gut diseases such as colon cancer, inflammatory bowel diseases, and irritable bowel syndrome has greatly increased, with possible new therapeutic strategies. More surprisingly, gut dysbiosis has been implicated in chronic metabolic disorders such as obesity, metabolic syndrome, diabetes, and cardiovascular diseases [3]. Nonalcoholic fatty liver disease (NAFLD) is the liver manifestation of the metabolic syndrome and thus evolves in the same context as these metabolic diseases [4]. It is therefore not surprising that recent literature emphasizes a potential role for gut dysbiosis in the pathophysiology of NAFLD.

NAFLD encompasses a spectrum of hepatic pathology (i.e., liver phenotypes). Accumulation of triglycerides in hepatocytes (hepatic steatosis) is the most common liver phenotype in NAFLD. Some individuals with hepatic steatosis develop nonalcoholic steatohepatitis (NASH), a more severe type of liver damage characterized by hepatic inflammation and liver cell death. In some individuals with the NASH phenotype, liver regeneration cannot keep pace with the increased rate of hepatocyte death, and liver scarring (fibrosis) ensues. Over time, some of these individuals accumulate sufficient fibrosis to develop cirrhosis. Because cirrhosis dramatically increases the risk for both primary liver cancer and overall liver-related mortality, liver cirrhosis is the NAFLD phenotype that has the worst prognosis. Epidemiologic studies indicate that NAFLD is now the most common cause of liver disease in many countries, including the United States [5]. It is estimated that at least 25% of American adults have some form of NAFLD, with about 6% of the general adult population having NASH and 2%–3% having NAFLD-related cirrhosis.

## Human Association Studies Suggesting a Link between Gut Microbiota and NAFLD Severity

The first evidence that gut dysbiosis might be involved in NAFLD pathogenesis was provided by a few cross-sectional studies that evaluated the association between gut microbiota and the liver phenotype in NAFLD patients. Using quantitative polymerase chain reaction (qPCR) for selected bacteria in a small cohort of 50 patients (17 controls, 11 patients with hepatic steatosis, and 22 patients with NASH), Mouzaki et al. showed that NASH patients had decreased fecal Bacteroidetes and increased *Clostridium coccoides* [6]. The negative association between NASH and Bacteroidetes persisted after adjustment for body mass index (BMI) and daily fat intake.

Zhu et al. screened the whole gut microbiota using 16S ribosomal RNA pyrosequencing in a pediatric cohort of 63 children that included 16 healthy controls, 25 obese subjects without known liver disease, and 22 patients with biopsy-proven NASH [7]. They found that fecal species richness was diminished in obese subjects and NASH patients compared to controls. Most samples clustered by health status but not by age, gender, or ethnicity, indicating a specific connection between the liver phenotype and gut microbiome. At the phylum level, obese patients and NASH patients had a similar increase in Bacteroidetes and decrease in Firmicutes compared to controls. Proteobacteria exhibited a progressive increase from the control to the obese and the NASH groups and was the only phylum with a significant difference between obese and NASH patients. The decreased representation of Firmicutes in the obese and the NASH groups was mostly explained by a decreased abundance in two families: Lachnospiraceae and Ruminococcaceae, with the greatest reduction for *Blautia* and *Faecalibacterium* genera. The increase in Proteobacteria was mainly explained by an increased abundance of Enterobacteriaceae, especially *Escherichia*, which was the only abundant genus within the whole bacteria domain exhibiting a significant difference between the obese and the NASH groups. Interestingly, Escherichia are known alcohol-producing bacteria, and serum alcohol concentration was significantly higher in NASH patients compared to obese or control groups.

The Mouzaki and Zhu studies may seem quite conflicting, the first showing a decrease in Bacteroidetes and the second an increase. However, different populations were studied (adults versus children, controls: biopsy-proven steatosis versus obese patients without histology) using different approaches (qPCR versus pyrosequencing). Both works were also limited by small sample size. Thus, further studies including larger and well-characterized cohorts are required to better identify the associations between gut microbiota and the various liver phenotypes observed in NAFLD.

## Animal Mechanistic Studies Demonstrating an Implication of Gut Microbiota in NAFLD

Cross-sectional studies allow for the discovery of potential associations between liver phenotype and certain gut bacteria, but they cannot establish a causal link. By using gut microbiota manipulations, recent animal studies have demonstrated direct roles for gut microbiota in each liver lesion observed in NAFLD: steatosis, NASH, fibrosis, and liver cancer.

### Liver steatosis

Conventional C57BL/6J mice fed with a high-fat diet (HFD) for 16 weeks generally display liver steatosis, hyperglycemia, and systemic inflammation (responders), but some mice are nonresponders, developing no metabolic disorder with this dietary manipulation [8]. To explore the potential role of gut microbiota in these discrepant responses, gut microbiota from a responder or from a nonresponder mouse were transplanted into germ-free mice (i.e., responder or nonresponder-receivers) that were then fed a HFD for another 16 weeks. Despite similar weight gain, responder-receiver mice developed a higher level of liver steatosis, glycemia, and insulin resistance than nonresponder-receivers. These data support the concept that interindividual differences in the intestinal microbiome modulate the metabolic and hepatic consequences of high-fat-diet consumption. Potential mechanisms for enhanced liver steatosis were demonstrated in earlier studies. The intestinal microbiome can increase the efflux of free fatty acids to the liver by influencing intestinal expression of the lipoprotein lipase inhibitor Fiaf (fasting-induced adipose factor, also known as angiopoietin-like protein-4) [9, 10]). Colonic bacteria also ferment nondigestible carbohydrates to short-chain fatty acids (SCFAs). SCFAs have been proposed to contribute to obesity and liver steatosis as they provide approximately 10% of daily caloric consumption and may enhance nutrient absorption by promoting expression of glucagon-like peptide 2 [11, 12]. However, SCFAs also improve lipid and glucose metabolism and maintain intestinal homeostasis [11, 13]. Hence, the net effect of SCFAs on NAFLD pathogenesis remains unclear and is likely complex. For example, although total cecal SCFA concentrations of recipient mice given flora from responder versus nonresponder mice were similar in the Leroy study, two branched-chain fatty acids (isobutyrate and isovalerate) were significantly higher in responder-receiver mice. Branched-chain fatty acids, which can be de novo synthetized by several gut bacterial species, have been associated with insulin resistance and metabolic disease development [14].

### Nonalcoholic steatohepatitis

Interindividual differences in the intestinal microbiome and severity of NASH, a more serious NAFLD phenotype, have also been linked. Targeted disruption of the NLRP3 or NLRP6 inflammasome altered the gut microbiota and was associated with enhanced colonic inflammation and NASH in mice fed methionine-choline-deficient-diets [15]. By studying several knockout models, the researchers discovered that more severe diet-induced NASH resulted from influx of intestinally derived toll-like receptor 4 (TLR4) and toll-like receptor 9 (TLR9) agonists into the portal circulation, which, in turn, activated tumor necrosis factor alpha (TNFα) in the liver. Wild-type mice that were cohoused with inflammasome-deficient mice also exhibited worsened NASH. Antibiotic treatment with ciprofloxacin and metronidazole reduced the severity of NASH in inflammasome-deficient mice and abolished transmission of the phenotype to wild-type animals, showing that gut microbiota drive NASH progression in this model. These findings have clinical relevance because human studies have demonstrated that NASH patients have greater endotoxemia and higher liver TNFα levels than patients with simple hepatic steatosis [16–18]. However, endotoxemia does not seem to be an absolute requirement for NASH development, as it was absent in a majority of patients in another NASH cohort [19].

### Liver fibrosis

Gut microbiota can also promote liver fibrosis, a known risk factor for NAFLD-related cirrhosis. In a recent study, mice fed a HFD before bile duct ligation (BDL) developed more severe liver fibrosis than control mice that were fed a standard chow diet before BDL [20]. HFD-related increases in liver fibrosis were associated with gut dysbiosis, especially an increase in Proteobacteria. To establish the causal link between the gut dysbiosis and worsened liver fibrosis, gut microbiota transplantation was performed before BDL. Control mice that received the gut microbiota from HFD mice demonstrated more severe liver fibrosis after BDL than HFD mice that were transplanted with gut microbiota from chow-fed mice. Selective transplantation of gram-negative or gram-positive bacteria showed that gram-negative bacteria were responsible for the observed enhancement of liver fibrosis.

### Liver cancer

A recent study established a link between the gut microbiota and NAFLD-related hepatocellular carcinoma [21]. Neonatal mice were treated with a single application of the carcinogenic agent dimethylbenz(a)antracene and then fed a HFD or standard chow diet for 30 weeks. HFD-fed mice developed more hepatocellular carcinoma, and intestinal decontamination with antibiotics reduced liver cancer formation. Metabolomic analysis showed that HFD feeding increased deoxycholic acid, a secondary bile acid generated solely by gut bacterial strains that 7α-dehydroxylate primary bile acids (e.g., Clostridium cluster XI and XIVa). Oral antibiotics decreased deoxycholic acid levels in HFD-fed mice. Moreover, deoxycholic acid administration restored liver cancer development in HFD-fed mice despite concomitant treatment with antibiotics. These results demonstrate the importance of the gut microbiome in modulating bile acid homeostasis. However, the relationship between bile acids and severity of NAFLD is very complex, with deleterious or beneficial effects probably depending on the type of bile acid and/or the particular bile-acid-sensitive signaling pathway targeted. Bile acids are ligands for the farnesoid X receptor (FXR) and the G-protein-coupled receptor TGR5, both having been implicated in metabolic syndrome pathobiology [22, 23]. In a recent randomized double-blind placebo-controlled phase 2 clinical trial, obeticholic acid, a semisynthetic FXR agonist derived from the primary human bile acid chenodeoxycholic acid, significantly improved markers of liver inflammation and fibrosis in NAFLD patients with type 2 diabetes [24]. Thus, both harmful and beneficial hepatic effects of bile acids have been demonstrated in NAFLD, and further research is necessary to clarify the basis for the discrepant outcomes.

## Gut Microbiota—Environment Interactions

There are thousands of bacterial species in the gut, and they all display an incredibly wide range of metabolic functions. Now that we realize that gut microbiota modulate NAFLD-related pathophysiology, the challenge is to decipher the mechanisms by which they exacerbate NAFLD severity [25]. As mentioned previously, gut microbiota could drive the severity of NAFLD through increased endogenous production of alcohol, activation of TLR signaling and TNFα production in the liver, or by altering the bile acid profile. Other potential mechanisms are emerging rapidly. For example, the intestinal microbiome seems to play an important role in regulating the availability of dietary choline, and choline deficiency is well-known to cause NASH with fibrosis in rodents [26]. A metabolomic study in C129S6 mice showed that feeding a HFD shifts the gut microbiota into a choline-degradation profile, resulting in low circulating levels of plasma phosphatidylcholine and high urinary excretion of methylamines [27]. The reduced bioavailability of choline in the HFD-fed mice mimicked the effect of choline-deficient diets that cause NAFLD. This information is clinically relevant because a recent human study demonstrated increased hepatic steatosis in 15 women who ate low-choline diets for 42 days [28]. Sequencing gut microbiota before the diet identified two classes of bacteria that predicted choline-deficiency-induced fatty liver: Firmicutes/Erysipelotrichi and Proteobacteria/Gammaproteobacteria. Interestingly, taking into account microbiota profile and the polymorphism of phosphatidyl ethanolamine N-methyltransferase (PEMT), which encodes an enzyme in the choline biosynthetic pathway, improved the prediction of liver steatosis occurrence. These findings demonstrate that host genetic background, diet, and the gut microbiota interact to influence NAFLD pathogenesis in humans, as is now known to occur in rodent models.

NAFLD is associated with a higher prevalence of gastro-oesophageal reflux [29] that requires treatment such as proton pump inhibitors or histamine receptor 2 antagonists. These antisecretory therapies have been associated with modifications in the gut microflora composition and increasing risk of enteric infections [30]. The significance of antisecretory-treatment-induced gut dysbiosis on the course and the severity of NAFLD remains to be determined.

In summary, NAFLD is an extremely common, complex disease that results from interactions between susceptible polygenic backgrounds and environmental factors. Recent evidence has introduced gut microbiota as a new crucial player in this complex story. Deciphering the mechanisms linking gut microbiota to NAFLD and its severity will advance understanding of the disease’s pathogenesis, thereby identifying new therapeutic targets that will ultimately improve the outcome in patients with this disease.
